# *Pseudomonas aeruginosa* Outer Membrane Vesicles Triggered by Human Mucosal Fluid and Lysozyme Can Prime Host Tissue Surfaces for Bacterial Adhesion

**DOI:** 10.3389/fmicb.2016.00871

**Published:** 2016-06-03

**Authors:** Matteo M. E. Metruccio, David J. Evans, Manal M. Gabriel, Jagath L. Kadurugamuwa, Suzanne M. J. Fleiszig

**Affiliations:** ^1^School of Optometry, University of CaliforniaBerkeley, CA, USA; ^2^College of Pharmacy, Touro University CaliforniaVallejo, CA, USA; ^3^Alcon Research, Ltd., Johns CreekGA, USA; ^4^Alcon Research, Ltd., Fort WorthTX, USA; ^5^Graduate Groups in Vision Science, Microbiology, and Infectious Diseases and Immunity, University of CaliforniaBerkeley, CA, USA

**Keywords:** *Pseudomonas aeruginosa* outer membrane vesicles, human tears, lysozyme, cornea, epithelium, adhesion, microbial keratitis

## Abstract

*Pseudomonas aeruginosa* is a leading cause of human morbidity and mortality that often targets epithelial surfaces. Host immunocompromise, or the presence of indwelling medical devices, including contact lenses, can predispose to infection. While medical devices are known to accumulate bacterial biofilms, it is not well understood why resistant epithelial surfaces become susceptible to *P. aeruginosa*. Many bacteria, including *P. aeruginosa*, release outer membrane vesicles (OMVs) in response to stress that can fuse with host cells to alter their function. Here, we tested the hypothesis that mucosal fluid can trigger OMV release to compromise an epithelial barrier. This was tested using tear fluid and corneal epithelial cells *in vitro* and *in vivo*. After 1 h both human tear fluid, and the tear component lysozyme, greatly enhanced OMV release from *P. aeruginosa* strain PAO1 compared to phosphate buffered saline (PBS) controls (∼100-fold). Transmission electron microscopy (TEM) and SDS-PAGE showed tear fluid and lysozyme-induced OMVs were similar in size and protein composition, but differed from biofilm-harvested OMVs, the latter smaller with fewer proteins. Lysozyme-induced OMVs were cytotoxic to human corneal epithelial cells *in vitro* and murine corneal epithelium *in vivo*. OMV exposure *in vivo* enhanced Ly6G/C expression at the corneal surface, suggesting myeloid cell recruitment, and primed the cornea for bacterial adhesion (∼4-fold, *P* < 0.01). Sonication disrupted OMVs retained cytotoxic activity, but did not promote adhesion, suggesting the latter required OMV-mediated events beyond cell killing. These data suggest that mucosal fluid induced *P. aeruginosa* OMVs could contribute to loss of epithelial barrier function during medical device-related infections.

## Introduction

Contact lenses are among the indwelling medical devices that can promote bacterial-induced pathology. In addition to sight-threatening corneal infection, microbial contamination of lenses and lens cases can cause various potentially serious and/or painful inflammatory events including contact lens-related acute red eye (CLARE), contact lens-induced peripheral ulcer (CLPU), and infiltrative keratitis (IK) (Stapleton et al., 2009; Willcox et al., 2011; Nagaraju et al., 2014).

*Pseudomonas aeruginosa* has been the most common cause of contact lens-related corneal infection since soft contact lenses were introduced in the early 1970’s (Stapleton and Carnt, 2012). Why the normally resistant cornea becomes susceptible to this pathogen during lens wear is not well understood (Evans and Fleiszig, 2013; Mantelli et al., 2013). In the absence of lens wear, overcoming barrier function to *P. aeruginosa* requires significant compromise; e.g., superficial injury using tissue paper followed by ethylene glycol tetra-acetic acid (EGTA) treatment or profound innate immune deficiency (Mun et al., 2009; Alarcon et al., 2011; Augustin et al., 2011; Tam et al., 2011; Sullivan et al., 2015). Yet we have shown that the same bacteria can reliably infect the cornea in a (rat) lens-wearing model if given sufficient time, even if very small inocula are used (Tam et al., 2010). Since lenses removed from infected eyes caused disease faster than lenses inoculated *in vitro*, the data suggested that bacteria launched additional virulence factors when adapted to the eye-lens environment.

Under various growth conditions, *P. aeruginosa* and other Gram-negative bacteria, can release vesicles from their outer membrane (outer membrane vesicles, OMVs; Kadurugamuwa and Beveridge, 1995). OMVs can elicit a wide array of functions that can potentially influence bacterial survival and pathogenesis. For example, they are involved in bacterial responses to envelope or oxidative stress (Macdonald and Kuehn, 2013), competition in microbial communities (Kadurugamuwa and Beveridge, 1996), horizontal gene transfer (Rumbo et al., 2011) and host-pathogen interactions. OMVs can be found in clinical specimens from infected subjects and they can mediate immune suppression or immune stimulation (Namork and Brandtzaeg, 2002; Bauman and Kuehn, 2006; Tan et al., 2007). The mechanism by which OMVs exert these effects can involve delivery of their contents across anatomical barriers by fusion with host cell membranes (Kadurugamuwa and Beveridge, 1998), providing the potential to modulate numerous host cell functions (Yu and Kim, 2012). While the Gram-negative *Treponema denticola* OMVs have been shown to disrupt barrier function of cell monolayers in culture (Chi et al., 2003), whether OMVs can disrupt barrier function *in vivo* has not been explored for any bacteria.

Outer membrane vesicles shedding is a tightly regulated process, with different environmental stimuli or bacterial lifestyles able to produce different types of OMV in both appearance and content (Kadurugamuwa and Beveridge, 1995; Schooling and Beveridge, 2006; Maredia et al., 2012; Toyofuku et al., 2012; Park et al., 2014). In particular, bacteria can produce OMVs in response to stresses such as temperature (Katsui et al., 1982), oxidative stress (Thompson et al., 1985), and antibiotics (Kadurugamuwa and Beveridge, 1995), through activation of the SOS response (Maredia et al., 2012).

Known *P. aeruginosa* OMV contents include proteases involved in basement membrane traversal by *P. aeruginosa* (Alarcon et al., 2009), and Cif [Cystic Fibrosis Transmembrane-conductance Regulator (CFTR) Inhibitory Factor] toxin (Bomberger et al., 2009) which can remove CFTR from the membrane of host cells and target it for degradation (Bomberger et al., 2009, 2011). OMVs also represent an intrinsic component of *P. aeruginosa* biofilms (Schooling and Beveridge, 2006; Toyofuku et al., 2012), and are known to mediate bacterial co-aggregation to enable biofilm formation and colonization of receptive surfaces in *P. aeruginosa* (Beveridge et al., 1997; Schooling and Beveridge, 2006; Schooling et al., 2009) as well as other Gram-negative bacteria (Yonezawa et al., 2009).

Since biofilms can form on contact lenses *in vivo* (Tam et al., 2010) and in contact lens storage cases (Stapleton and Dart, 1995), and antimicrobials and other potential stressors can be present in either location, *P. aeruginosa*-derived OMVs could be relevant to the pathogenesis of contact lenses-related adverse events. Here, we tested the hypothesis that human tear fluid, its abundant antimicrobial constituents lysozyme and lactoferrin, or lysates of human corneal epithelial cells induce OMV production. We also explored if *P. aeruginosa*-derived OMVs could compromise corneal epithelial barrier function.

## Materials and Methods

### Human Tear Fluid

Human tears were collected from healthy human volunteers using a 30 μl-volume capillary tube under a protocol approved by the Committee for the Protection of Human Subjects, University of California, Berkeley. Subjects were non-contact lens wearers, both males and females, between 18 and 45 years of age and with no ocular infection or inflammation at the time of collection. Approximately 5% of the tear fluid from each subject was plated on TSA plates (see methods) to control for bacterial contamination; sterile tear fluid was pooled (from 6 to 8 subjects) and stored at -80°C until used.

### Reagents

Contrived artificial tears were purchased from Ursa BioScience (Bel Air, MD). The complete list of tear components, and their concentration, in the contrived artificial tears remains proprietary. However, the components disclosed by the manufacturer include immunoglobulins (γ-globulins), albumin (human), lysozyme and salts, with an osmolarity of 300 mOsm, and pH 7.4. Lactoferrin (from human milk), recombinant human lysozyme, and other reagents were purchased from Sigma–Aldrich (St. Louis, MO, USA).

### Cell Culture and Cell Lysates

Human telomerase immortalized corneal epithelial cells (hTCEpi; Robertson et al., 2005) were maintained at 37°C in 5% CO_2_ in keratinocyte growth medium (KGM-2) containing 0.15 mM CaCl_2_ (Lonza, Walkersville, MD, USA). Cells were grown as monolayers or multilayers on either 96-well tissue culture plates or airlifted on collagen-coated Transwell filters in 12-well tissue culture plates, for use in cytotoxicity and traversal experiments, respectively (see methods below). To prepare lysates from hTCEpi cells for treatment of bacteria, hTCEpi cells were grown on 6-well tissue culture plates until confluent, then lysed by repeated cycles of cell freezing and thawing in phosphate buffered saline (PBS) followed by centrifugation (12,000 × *g*, 10 min, 4°C) to remove insoluble material. The supernatant (lysate) was assessed for protein concentration [Bicinchoninic Acid (BCA) Assay Kit, Pierce Biotechnology Inc., Thermo Scientific] and exposed to *P. aeruginosa* at 0.5 or 1.0 mg/mL of total protein as outlined below.

### Bacterial Strains and OMV Preparation

*Pseudomonas aeruginosa* strains used were; PAO1, PA14 and 19660 (laboratory strains) and PAK, 6206 and 6294 (clinical isolates). For fluorescent imaging PAO1 was transformed with plasmid pSMC2 encoding green fluorescent protein (PAO1-GFP; Bloemberg et al., 1997) or d-Tomato fluorescent protein (Singer et al., 2010). Bacteria were grown on TSA plates for 24 h at 37°C except for plasmid-complemented bacteria for which TSA was supplemented with carbenicillin 400 μg/mL. For use in experiments, bacteria were resuspended into either PBS to a concentration of ∼10^11^ CFU/mL for OMV preparation, or in KGM-2 (without antibiotics) to ∼10^11^ CFU/mL then diluted as needed for use in *ex vivo* bacterial association, cytotoxicity or traversal experiments.

For preparation of OMVs from agar plate grown bacteria, a modified version of the protocol reported by Schooling and Beveridge (2006) was used. Briefly, *P. aeruginosa* strains were grown on 2 × 10 cm TSA plates for 24 h at 37°C to form a “lawn” covering. Bacteria were scraped from the agar surface and resuspended in 10 mL of PBS to form a homogeneous suspension of ∼10^10^ CFU/mL. Bacteria were then removed by centrifugation (12,000 × *g*, 20 min, 4°C) three times. Supernatants were then pooled, centrifuged (12,000 × *g*, 20 min, 4°C) and filtered (0.45 and 0.22 μm pore size) to remove remaining bacteria and large aggregates, and subjected to ultracentrifugation (150,000 × *g*, 3 h, 4°C). The pellet containing OMVs was then resuspended in PBS, and subjected to low speed centrifugation (16,000 × *g* for 30 min, 4°C) to clear most contaminating flagella (Renelli et al., 2004; Schooling and Beveridge, 2006). The resulting preparations were considered TSA-grown OMVs.

For induction of OMVs, PAO1 was treated with human tear fluid, contrived artificial tears, lysozyme, lactoferrin, or corneal epithelial cell lysates. Firstly, bacteria were grown overnight on TSA plates (as above), and then resuspended in 10 mL of PBS. After washing once to remove previously formed OMVs, bacteria were resuspended in 1 mL of PBS to a concentration of ∼10^11^ CFU/mL and incubated for 1 h at 37°C (uninduced OMVs). Otherwise, bacteria were resuspended in 1 mL of either human tear fluid (pooled from 6 to 8 subjects), lysozyme in PBS (1.5 mg/mL, 102 μM), lactoferrin in PBS (1 mg/mL, 13 μM), or corneal epithelial cell lysates (preparation described above), for 1 h at 37°C and compared with PBS (uninduced) controls. OMVs were then prepared as described above after dilution to 10 mL of PBS. The OMV preparations were quantified for total lipid content in triplicate using a fluorescent lipophilic dye FM1-43FX (Thermo Fisher Scientific; Frias et al., 2010; Murphy et al., 2014) according to manufacturer instructions. Relative fluorescence units (RFU)/mL was divided by optical density at 600nm (OD_600_) of the bacteria at the time of vesicle harvest to generate the OMV yield (RFU/OD_600_). The OMV preparations were also quantified for total protein content (BCA Assay Kit, Pierce). For SDS-PAGE, samples were either standardized to contain equivalent amounts of protein, or normalized according the number of bacteria used for OMV preparation (∼10^11^ CFU/mL). Samples were mixed with Laemmli buffer (Bio-Rad, Hercules, CA, USA), subject to electrophoresis using 4–15% Mini-Protean TGX Gels (Bio-Rad) and visualized with Coomassie blue or silver stain (Bio-Rad). In some experiments, lysozyme-induced OMVs were sonicated (6 cycles of 30 s each with 30 s pause at maximum power) on ice and/or proteinase K treated (5 μg/mL, 30 min, 37°C) to cause membrane disruption which was confirmed using SDS-PAGE stained with Coomassie blue.

### Transmission Electron Microscopy

Samples were assessed by negative staining as described by Schooling and Beveridge (2006), and examined using an FEI Tecnai 12 Transmission electron microscope operating at an acceleration voltage of 100 kV under standard operating condition.

### Assays for OMV Cytotoxicity and Bacterial Traversal

For cytotoxicity assays *in vitro*, hTCEpi cells in antibiotic-free KGM-2 media were grown to 90% confluence in 96-well tissue culture–treated plates and then incubated with intact lysozyme-induced OMVs, sonicated OMVs or uninduced (basal) OMVs for 1, 2, and 4 h. Each OMV sample was inoculated at a concentration corresponding to the approximate amount produced by ∼10^9^ CFU of *P. aeruginosa*. Cytotoxicity was calculated by measuring LDH release in the supernatant using a cytotoxicity detection kit (Roche, Indianapolis, IN, USA). In each experiment, percent cytotoxicity was calculated relative to PBS buffer as a negative control (0% cytotoxicity) and Triton X-100 (1% vol/vol) treated as a positive control (100% cytotoxicity). To asses cytotoxicity of OMVs in murine corneas, mice were anesthetized with intraperitoneal injection of ketamine (90 mg/kg) and dexmedetomidine (0.5 mg/kg; Hahn et al., 2005), then inoculated once with 5 μl of purified intact lysozyme-induced OMVs, sonicated lysozyme-induced OMVs, or PBS buffer, then incubated for 4 h. Anesthesia was maintained throughout the 4 h exposure period. OMV preparations were both at a concentration of ∼1 mg/mL of total proteins, corresponding to the approximate amount produced by ∼10^9^ CFU of *P. aeruginosa* strain PAO1, a typical inoculum size (in 5 μl) for studies of *P. aeruginosa* interactions with the murine cornea *in vivo* or *ex vivo* (Alarcon et al., 2011; Tam et al., 2011; Sullivan et al., 2015). After euthanasia, freshly enucleated eyes were washed three times in PBS prior to incubation with NucBlue (all cell nuclei) and NucGreen (permeabilized cell nuclei; Thermo Fischer) in DMEM (Gibco) at room temperature for 30 min. After rinsing, eyes were glued on coverslip, soaked in DMEM and imaged by confocal microscopy using reflection 635 nm (corneal cells), 405 nm (NucBlue) and 488 nm (NucGreen) lasers. Z stacks (2.0 μm steps) were collected from 4 or more eyes per treatment. Image analysis was performed with Image-J.

For *in vitro* bacterial traversal assays, polarized multilayers of hTCEpi cells were generated by seeding 5 × 10^4^ cells/mL onto 12-well collagen-coated tissue culture inserts (3 μm pore size; Corning, Inc., NY) in KGM-2 medium containing 1.15 mM CaCl_2_ (high calcium) and airlifting for 7 days as previously described (Robertson et al., 2005). Cells where then pre-incubated in the apical compartment for 1 h with intact (lysozyme-induced) OMVs, sonicated lysozyme-induced OMVs, uninduced (basal) OMVs or PBS buffer in 5% CO_2_ at 37°C. As with other assays, each OMV sample was inoculated at a concentration corresponding to the approximate amount produced by ∼10^9^ CFU of *P. aeruginosa*. After washing, PAO1 inocula (10^6^ CFU per well) in KGM-2 without antibiotics were carefully added to the apical compartment, and incubated with the cells (5% CO_2_ at 37°C) for 4 h. Traversed bacteria were then quantified by viable counts of the basal compartment.

### Cornea Whole Mount and Immunostaining

Eyes from C57BL/6 mice (male and female, 6–12 weeks of age) were used. The use of animals was approved by the Animal Care and Use Committee of the University of California, Berkeley. Following euthanasia, eyes were enucleated and fixed in 2% paraformaldehyde (PFA) for 15 min. The corneas were excised and further incubated in 2% PFA for an additional 45 min, washed and blocked in PBS/2% BSA for 30 min and permeabilized in PBS/2% BSA/0.1% TritonX-100 for 30 min at room temperature. Anti-Ly6G/C (NIMP-R14, Rat IgG2b, 1:50 Thermo Fisher) antibodies were prepared in the permeabilizing solution and incubated with the mouse corneas overnight at 4°C. The corneas were then washed with PBS, incubated with 488-Dylight anti-rat secondary antibody (Thermo Fisher) for 3 h at room temperature, and counterstained with DAPI. The whole mount corneas were cover slipped and visualized using confocal microscope as described above. As a control, some samples were processed without primary antibody to control for non-specific binding of secondary antibody, or using an isotype control (Rat IgG2b, 1:50 Thermo Fisher).

### Bacterial Association with Murine Corneal Epithelium *Ex Vivo*

3D quantitative confocal imaging *ex vivo* was used as previously described (Tam et al., 2011) with some minor modifications. Briefly, enucleated eyeballs of C57BL/6 wild-type mice were rinsed with PBS, untreated or blotted with tissue paper on the corneal surface to enable susceptibility to bacterial adhesion (Alarcon et al., 2011), and pre-incubated for 1 h with the following: purified intact lysozyme-induced OMVs, sonicated lysozyme-induced OMVs, uninduced (basal) OMVs or PBS buffer. Preparations were standardized according to the number of bacteria used to generate the OMV preparations (∼10^11^ CFU/mL). In other experiments, non-blotted eyes were inoculated *in vivo* with purified intact lysozyme-induced OMVs, sonicated lysozyme-induced OMVs or PBS buffer for 4 h under anesthesia prior to sacrificing the mice and enucleating the eyes as described above (see Assays for OMV cytotoxicity and traversal). Eyeballs were then incubated at 37°C in ∼10^11^ CFU/mL of *P. aeruginosa* PAO1-GFP for 5 h. After rinsing to remove non-adherent bacteria, eyes were placed on cotton wool soaked in Dulbecco’s Modified Eagle’s Medium (DMEM) and imaged by confocal microscopy with unpreserved eye drop as an interface with the objective lens, using reflection 635 nm (corneal cells), 559 nm (PAO1-dTomato) 488 nm (PAO1-GFP or NucGreen) and 405 (NucBlue/DAPI) lasers. Z stacks (1.0 μm steps) were collected from 4 or more random fields per sample. 3-D image reconstruction was performed by Image-J. Bacterial adherence was defined as the total area (μm^2^) occupied by the GFP or dTomato signal on the corneal surface for each field.

### Statistical Analysis

*In vitro* data were expressed as a mean ± standard deviation (SD). Statistical analysis of differences between groups was determined using Student’s *t*-Test or ANOVA with Tukey’s *post hoc* analysis. For *ex vivo* experiments, data were expressed as a median with upper and lower quartiles, and differences between groups determined with ANOVA with Tukey’s *post hoc* analysis, or the Mann Whitney *U* test or Kruskal–Wallis test for two or three groups, respectively. *P*-values of < 0.05 were considered significant. All experiments were performed at least three times unless otherwise stated.

## Results

### *P. aeruginosa* Strains Able to Infect the Cornea Differ in OMV Quantity and Composition

**FIGURE 1:**
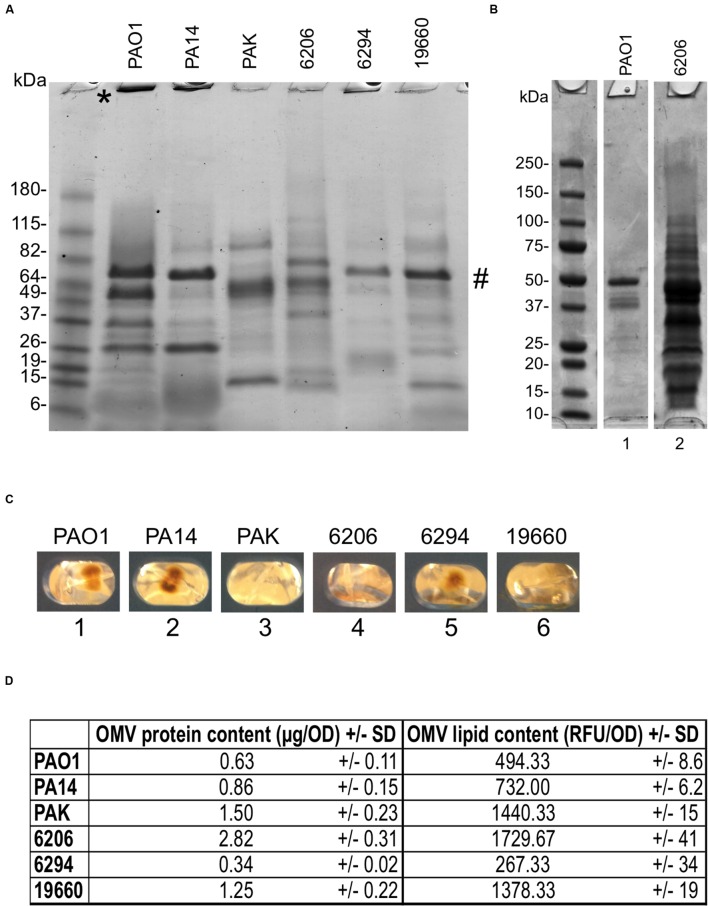
**(A)** SDS-PAGE of outer membrane vesicles (OMVs) purified from six different *Pseudomonas aeruginosa* strains showing relative differences in protein composition after 24 h growth on TSA at 37°C (TSA-grown OMVs). Samples were standardized to contain equal amounts of protein (∼10 μg). ^∗^ High molecular weight bands at the top of lanes. # Dominant band from pellets of 6294 and 19660 also found in PAO1 and PA14 **(B)** OMV preparations from strains 6206 and PAO1 grown as above, but normalized for bacterial numbers (see Materials and Methods). Under these growth conditions, strain 6206 showed greater OMV production (0.9 vs. 0.2 mg/mL of total protein). **(C)** Photographs of OMV pellets from different *P. aeruginosa* strains after ultracentrifugation and before resuspension with PBS show differences in OMV pellets between lanes 1, 2, and 5 (brown, gelatinous) vs. 3, 4, and 6 (clear). **(D)** Quantification of total protein content (μg/OD_600_) and lipid content (Relative Fluorescent Units, RFU/OD_600_) normalized to bacterial OD, for the six different *P. aeruginosa* isolates.

First, we explored the OMV forming capacity of six *P. aeruginosa* strains able to cause corneal infections in mice (Preston et al., 1995). They included three laboratory strains (PAO1, 19660, and PA14) and three clinical isolates from human infections (6206 and 6294, from corneal infections and strain PAK from bacteremia). To trigger OMV production, extended growth (24 h) on Trypticase Soy Agar (TSA) at 37°C was used (Schooling and Beveridge, 2006). While all strains produced OMVs, there was considerable variability in protein and lipid content for each (**Figures 1A,D**). For example, strain 6206 released more OMVs than strain PAO1 (2.82 vs. only 0.63 μg/OD_600_ of protein content, or 1729.67 vs. 494.33 RFU/OD_600_ of lipid content; **Figures 1B,D**). Greater amounts of high molecular weight bands at the top of lanes, e.g., PAO1 and PA14 (**Figure 1A**), suggested differences in OMV aggregation capabilities between strains. There were also differences in the appearance of OMV pellets after ultracentrifugation (**Figure 1C**). PAO1, PA14, and 6294 (lanes 1, 2, and 5) yielded a bigger, yellow, gelatinous pellet; while OMV pellets from PAK, 6206, 19660 (lanes 3, 4, and 6) were clear. These two phenotypes were reported previously when comparing OMV pellets from planktonically-grown PAO1 (clear) to those from biofilm grown PAO1 (bigger, brown and gelatinous; Schooling and Beveridge, 2006). Differences in pellet appearance did not seem to correlate with differences in protein banding patterns; a dominant band was observed in strains 6294 and 19660 (**Figure 1A**), which had gelatinous and clear pellets, respectively (**Figure 1C**), although the clear OMV pellets of 6206, PAK, 19660 showed more bands of higher MW (**Figure 1A**). There were also no obvious correlations of the two groups with previously described differences among these strains, e.g., ocular isolates, LPS serogroup, or cytotoxic versus invasive phenotypes. For the remainder of the study we used PAO1 because of the extensive genotypic and phenotypic characterization of this strain in the literature compared to the other strains.

### Human Tear Fluid and Lysozyme Induce OMV Release by PAO1

Under a contact lens, bacteria are exposed to the posterior surface of the lens, epithelial cells at the anterior surface of the cornea on which the lens sits, and tear fluid trapped between these two surfaces. Given the presence of antimicrobial factors associated with corneal epithelial cells and tear fluid, we tested both for their capacity to trigger OMV production by *P. aeruginosa* strain PAO1.

**FIGURE 2:**
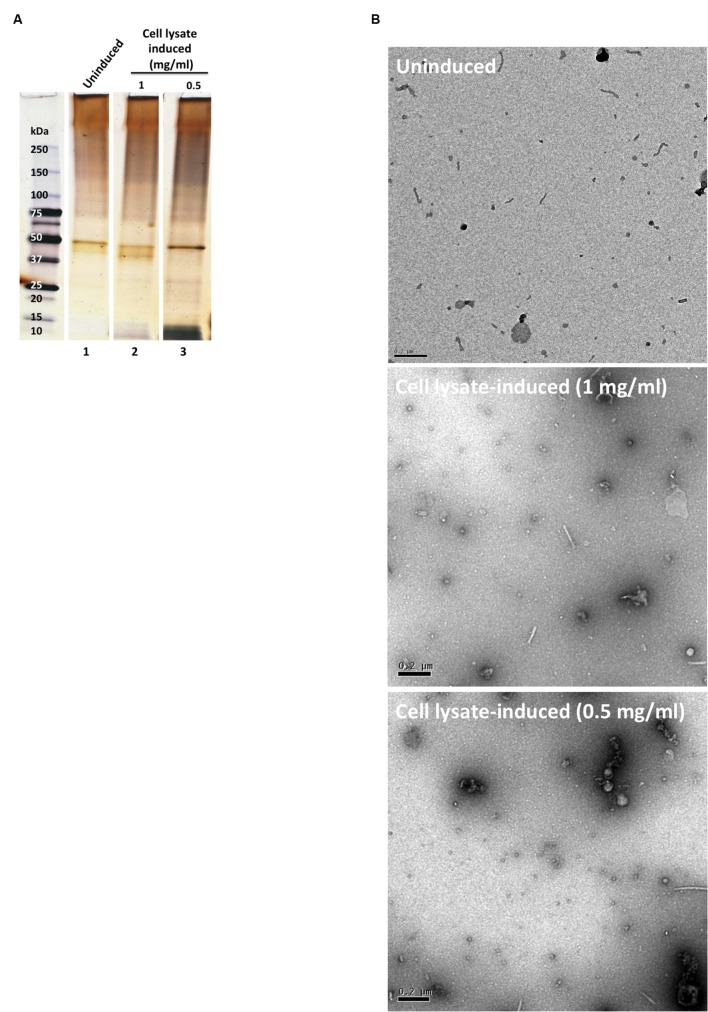
**(A)** SDS-PAGE silver-stained gel showing OMV preparations after 1 h incubation in PBS buffer (uninduced, lane 1) and corneal epithelial cell lysate induced (lanes 2 and 3). OMV preparations were normalized for bacterial numbers **(B)** TEM micrographs of the OMV preparations. Scale bar = 200 nm.

When the bacteria were exposed to cell lysates of cultured human corneal epithelial cells for 1 h (Cell lysate-induced), OMV release was similar to the baseline control (Uninduced) PBS exposure (**Figure 2A**). Transmission electron microscopy (TEM) micrographs confirmed that few or no OMVs were present in either the uninduced PBS or induced cell lysate bacterial samples (**Figure 2B**). The nature of the small round particles observed in the induced cell lysate samples (**Figure 2B**) was not clear; morphological appearance and protein banding pattern suggested they were not bacterial OMVs, but instead likely to be vesicles or debris derived from the epithelial cells.

**FIGURE 3:**
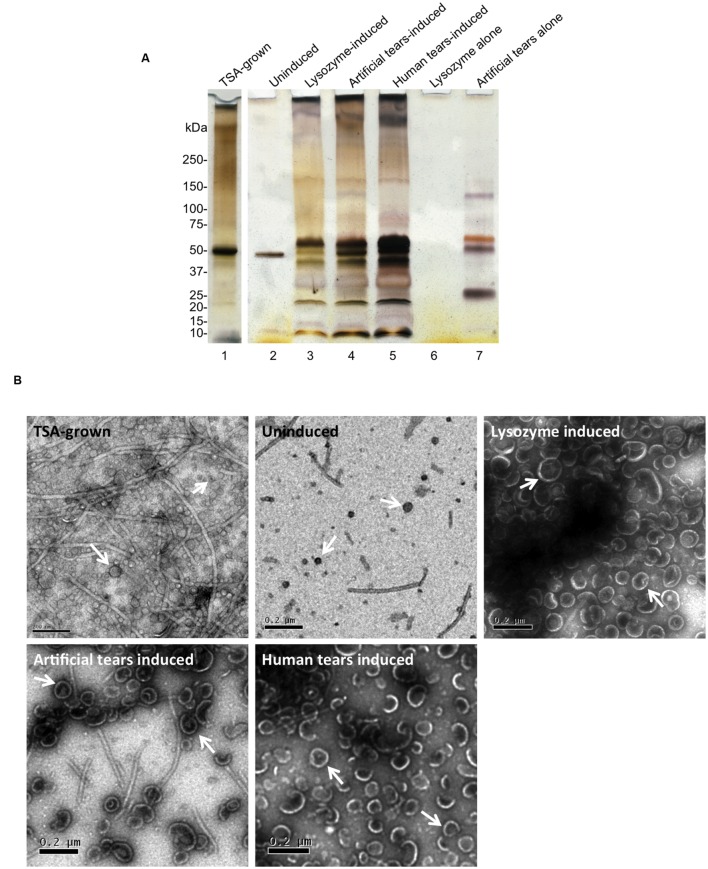
**(A)** SDS-PAGE (silver-stain) gels showing OMV preparations from *P. aeruginosa* strain PAO1 after 24 h growth on TSA (TSA-grown), 1 h incubation in PBS (uninduced), lysozyme (1.5 mg/mL), contrived artificial tear fluid, or human tear fluid. As controls, lysozyme or contrived artificial tears without bacteria were subject to OMV purification. Lysozyme did not carryover into OMV preparations after purification (lane 6). However, some components of contrived tears survived the OMV purification process (lane 7). TSA-grown indicates OMV extracted after 24 h growth on TSA. OMV preparations were normalized for bacterial numbers. However, protein concentration of TSA-grown OMVs (0.86 μg/μl) was similar to lysozyme-induced OMVs (1 μg/μl) allowing comparison of lanes 1 and 3 from that perspective. **(B)** TEM micrographs of OMVs, after 24 h growth on TSA (TSA-grown), 1 h treatment with PBS (uninduced), contrived artificial tear fluid, lysozyme and human tear fluid. Differences in OMV size and relative quantity were clearly evident along with some residual flagella contamination that may contribute to protein banding in **(A)**. Scale bar = 200 nm.

Next, bacteria were exposed to undiluted tear fluid collected from human volunteers or commercially available artificial human tear fluid for 1 h, which both triggered a large increase in OMV production compared to uninduced control as shown in **Figure 3A**, lane 2 vs. lanes 4 and 5 (∼100-fold increase as measured by total protein content, ∼60-fold increase as measured by total lipid content). To explore which tear components triggered OMVs we tested purified lactoferrin and lysozyme, selected because of their abundance in human tear fluid and artificial tears, their known antimicrobial activity (Selinger et al., 1979; Flanagan and Willcox, 2009), and because both can deposit on the posterior surface of contact lenses (Hall et al., 2014). Lysozyme, but not lactoferrin, triggered OMV release, and lysozyme-triggered OMVs had a similar protein-banding pattern to those induced by human tear fluid and contrived artificial tears (**Figure 3A**, lanes 3–5) and different protein banding patterns than OMVs extracted after growth on TSA for 24 h (**Figure 3A**, lane 1 vs. lanes 3–5). In this Figure, OMV preparations were normalized for bacterial numbers to show the impact of TSA growth versus tear fluid (or tear components) on the same number of bacteria. As such protein concentrations varied between OMV samples. However, the protein concentration of TSA-grown OMVs (0.86 μg/μl) was quite similar to lysozyme-induced OMVs (1 μg/μl) allowing a comparison of lanes 1 and 3 from that perspective. Controls without bacteria confirmed the absence of lysozyme carryover into OMV preparations after purification (**Figure 3A**, lane 6). However, some components of contrived artificial tears were retained after OMV purification (**Figure 3A**, lane 7). As expected, based on published literature (Cowell et al., 1997; Callewaert et al., 2008; Kamiya et al., 2012), neither human tear fluid, nor lysozyme, affected viability of *P. aeruginosa* strain PAO1 (measured by CFU viable counts before and after 1 h incubation) compared to untreated controls (data not shown). While artificial tears caused a small decrease in bacterial viability, it was not significantly different to untreated controls (*p* = 0.075, Student’s *t*-test).

Transmission electron microscopy (TEM) was used to assess morphological aspects of OMVs after the various induction methods. Representative micrographs are shown in **Figure 3B**. While the uninduced (PBS) control samples contained only a small number of OMVs, numerous OMVs were released after 24 h growth on TSA plates (TSA-grown OMVs), or 1 h treatment with either human tear fluid (induced OMVs) contrived artificial tears, or lysozyme. Qualitatively, OMVs induced by tear fluid or lysozyme appeared larger and more homogeneous than TSA-grown OMVs. There was also evidence of residual flagella contamination of OMV preparations, despite efforts to remove it with low-speed centrifugation, and this may contribute to protein banding patterns seen in **Figure 3A**. Differences observed in SDS-PAGE OMV banding patterns (**Figure 3A**) and morphology (**Figure 3B**) suggested that tear fluid or lysozyme may induce different OMVs from *P. aeruginosa* compared to bacteria grown for 24 h on TSA. However, further studies are needed to fully investigate this finding.

### Lysozyme-Induced PAO1 OMVs Are Cytotoxic to Human Corneal Epithelial Cells *In Vitro*

**FIGURE 4:**
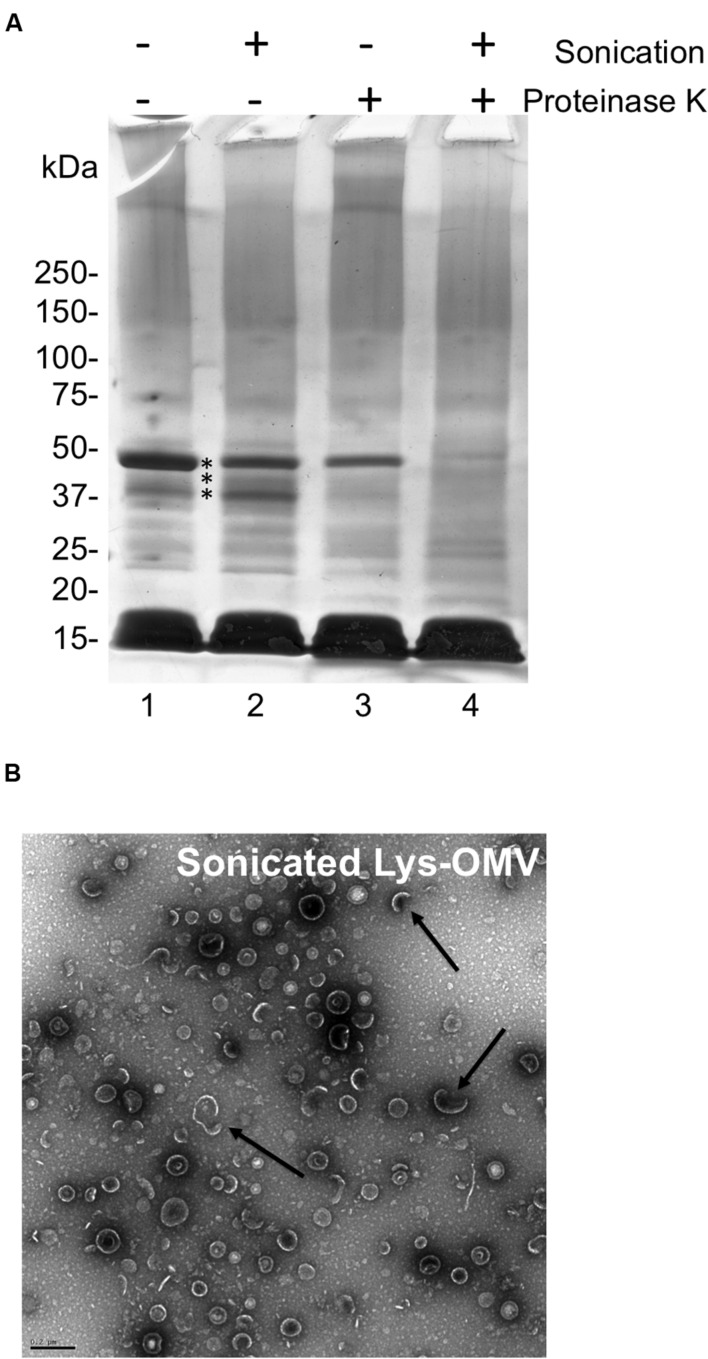
**(A)** SDS-PAGE with Coomassie stain showing *P. aeruginosa* PAO1 OMV preparations induced by purified human lysozyme (1.5 mg/mL) for 1 h with and without sonication or proteinase K treatment. Untreated lysozyme-induced OMVs (lane 1), sonicated lysozyme-induced OMVs (lane 2), proteinase K treated lysozyme-induced OMVs (lane 3), sonicated and proteinase K treated lysozyme-induced OMVs (lane 4). Comparison of the latter with lanes 1–3 indicated several proteins (asterisks) that became more susceptible to proteinase K digestion suggesting the disruption of OMV membrane integrity. Samples were standardized to contain equal amounts of protein (∼10 μg). **(B)** TEM micrograph of sonicated lysozyme-induced OMVs (Sonicated Lys-OMV) showing semi-disrupted vesicles (arrows). Scale bar = 200 nm.

To explore the impact of OMVs on corneal epithelial cells, lysozyme was used rather than human tear fluid or artificial tears due to; (1) the ready availability and consistency of purified human lysozyme compared to native human tears, (2) the similarity of OMVs obtained using lysozyme (**Figures 3A,B**), and (3) the absence of lysozyme (or other contaminant) carry-over from the OMV preparation protocol (**Figure 3A**, lane 6) compared to artificial tears (**Figure 3A**, lane 7). To distinguish between impact of intact OMVs and their contents, a portion of lysozyme-induced OMV preparations were sonicated to disrupt membrane integrity before each assay. Membrane disruption after sonication was verified using proteinase K digestion and TEM (**Figures 4A,B**).

**FIGURE 5:**
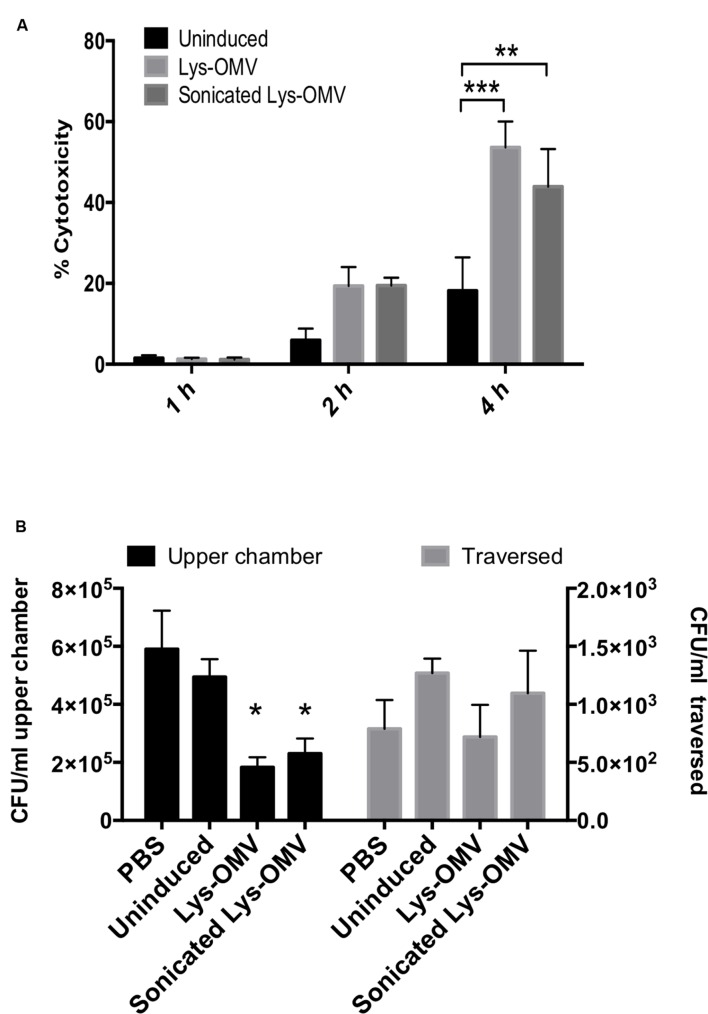
**(A)** Cytotoxicity of uninduced or lysozyme-induced OMVs (Lys-OMV) toward human corneal epithelial cells measured by LDH release. Percent cytotoxicity expressed relative to PBS buffer and Triton X-100 (1% vol./vol.) as negative and positive controls, respectively. **(B)** Effects of OMV pre-treatment on *P. aeruginosa* traversal of multilayered human corneal epithelial cells *in vitro*. After 1 h pre-incubation with PBS or different OMV preparations, airlifted cell multilayers were challenged with ∼10^6^ CFU of *P. aeruginosa* PAO1 for 4 h (5% CO_2_, 37°C). Bacteria were recovered and counted from the apical compartment (upper chamber, black bars, left *Y*-axis) and basal compartment (traversed, gray bars, right *Y*-axis). Significantly fewer bacteria were recovered from the apical (inoculation) compartment for lysozyme-induced OMVs (Lys-OMV) by the end of the assay; lysozyme-induced OMVs (intact or sonicated) had no impact on traversal. Data are expressed as the mean ± SD from three independent experiments. ^∗^*P* < 0.002 compared to PBS, ^∗∗^*P* = 0.0017, ^∗∗∗^*P* = 0.0002 (Two-way ANOVA with Tukey’s multiple comparison test).

To ensure relevance to humans, we first used cultured telomerase immortalized human corneal epithelial cells to examine the cytotoxic capacity of OMVs. Cells were grown as monolayers to ensure that the apical side of every cell in the culture would be exposed to OMVs. Cytotoxicity was quantified using LDH release assays. As shown in **Figure 5A**, lysozyme-induced OMVs were ∼3-fold more cytotoxic toward cultured human corneal epithelial cells than uninduced preparation, with greater than 50% cytotoxicity after 4 h of exposure. No cytotoxicity was observed after 1 h, and differences were not yet significant at 2 h. Sonicated OMVs killed a similar percentage of cells as intact OMVs, showing that OMV membrane integrity was not required for the cytotoxic effects of lysozyme-induced OMVs.

### Lysozyme-Induced OMV Pretreatment for 1 h Does Not Promote Susceptibility to *P. aeruginosa* Traversal for Multilayered Human Corneal Epithelial Cells Grown *In Vitro*

Multilayers of human corneal epithelial cells were then used to determine if lysozyme-induced OMVs had any impact on epithelial barrier function to *P. aeruginosa* at a time when OMVs were not yet cytotoxic to the cells (i.e., 1 h). Cells were grown on Transwell filters (3 μm pore-size) and subsequently airlifted for 7 days to encourage stratification before pre-treatment with PBS, uninduced preparation, or lysozyme-induced OMVs (intact or sonicated) for 1 h. After washing, ∼10^6^ CFU *P. aeruginosa* PAO1 were added to the apical cell surface (apical compartment) for 4 h before viable counting both the apical and basal chambers to enumerate survivors at the inoculation surface and those that had traversed the cells. The results revealed no significant difference in the number of bacteria that traverse the cells after the 1 h pretreatment with OMVs (**Figure 5B**, gray bars). Intriguingly, fewer bacteria were recovered from the apical (inoculation) compartment for lysozyme-induced OMVs by the end of the assay (**Figure 5B**, black bars), which might have masked increases in susceptibility to bacterial traversal for those samples.

### Lysozyme-Induced PAO1 OMVs Are Cytotoxic to Host Cells in the Mouse Cornea *In Vivo* and Promote Recruitment of Ly6G/C Positive Cells

**FIGURE 6:**
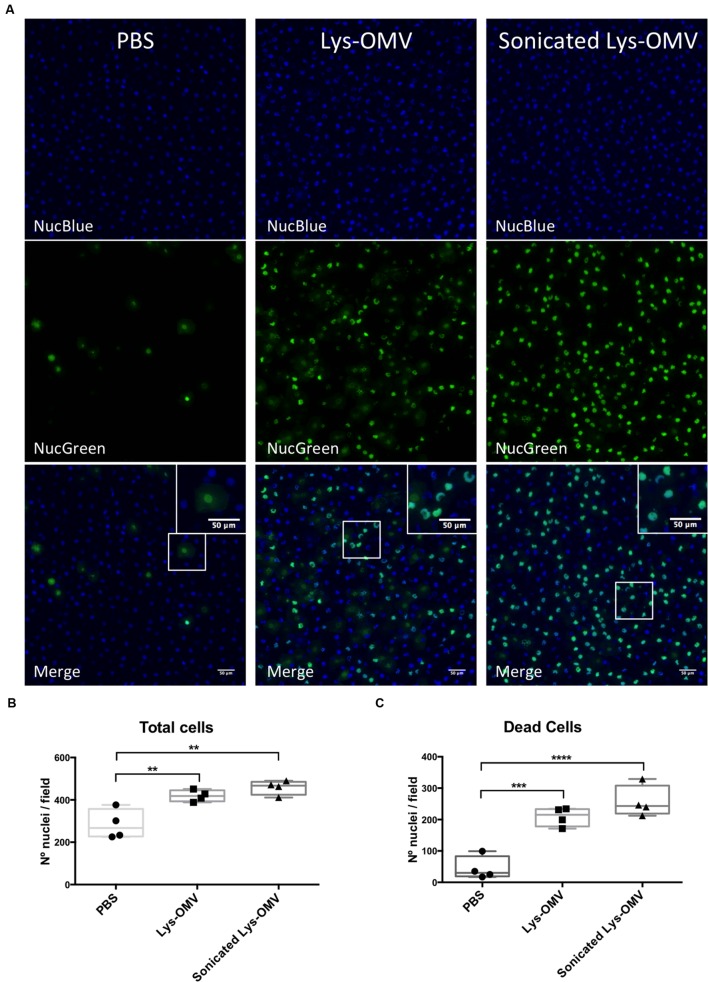
**(A)** Cytotoxicity in murine corneas after 4 h *in vivo* treatment with PBS, lysozyme-induced OMVs (Lys-OMV) and sonicated lysozyme-induced OMVs (1 mg/ml of total proteins). Whole eyes where enucleated and incubated with NucBlue (all nuclei) and NucGreen (nuclei of permeabilized cells) for 30 min prior to imaging. Maximum intensity projection of blue channel (all nuclei), green channel (dead cells) and merge from a representative experiment are shown for each condition. **(B,C)** Quantification of total cells and dead cells from four mice per group across four independent experiments. Data are expressed as a median with upper and lower quartiles. ^∗∗^*P* < 0.01, ^∗∗∗^*P* < 0.001, ^∗∗∗∗^*P* < 0.0001 (One-way ANOVA with Tukey’s multiple comparison test).

**FIGURE 7:**
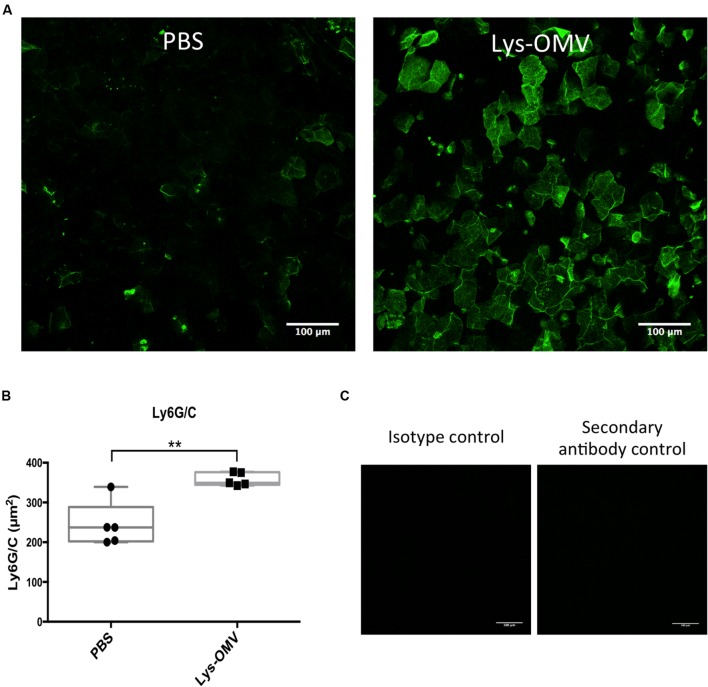
**(A)** Ly6G/C flat mount immune staining showing an increase in the number of cells expressing the myeloid marker Ly6G/C after a 4 h *in vivo* inoculation with lysozyme-induced OMVs (Lys-OMV) or PBS as a control. **(B)** Quantification of the Ly6G/C positive signal (green) in μm^2^ from five mice per group across three independent experiments. **(C)** Controls showing the absence of Ly6G/C labeling in OMV treated samples (4 h) after using an isotype antibody (Rat IgG2b) or a secondary antibody only. Data expressed as a median with upper and lower quartiles. ^∗∗^*P* < 0.008 (Mann–Whitney *U* test).

Mice were used to study the cytotoxicity of OMVs *in vivo*, utilizing NucGreen to visualize permeabilized corneal cells. As shown in **Figure 6A**, after 4 h *in vivo* treatment with lysozyme-induced OMVs, there was a consistent increase in the number of dead cells (green) compared to PBS inoculated corneas (quantified in **Figure 6C**). Consistent with the *in vitro* human cell data, we observed no differences in the number of dead cells for mouse corneas treated with intact and sonicated OMV preparations. Surprisingly, for both intact and sonicated OMV treated corneas we also observed a general increase in the number of nuclei positive for NucBlue (DAPI), a dye that labels all nuclei irrespective of their membrane permeability (**Figure 6B**). Likely contributing to this increase in cell number at the corneal surface, some cells in OMV treated corneas had irregular nuclei suggesting that they were myeloid cells, not epithelial cells (enlarged panels in **Figure 6A**, merge). Labeling of whole-mount mouse corneas for the cell marker Ly6G/C supported the hypothesis that these were myeloid cells recruited as a result of OMV challenge (**Figure 7A**, quantified in **Figure 7B**), the use of isotype and secondary-only antibody controls confirmed labelling specificity (**Figure 7C**).

### Lysozyme-Induced OMVs Promote *P. aeruginosa* Association with the Murine Corneal Epithelium *Ex Vivo*

**FIGURE 8:**
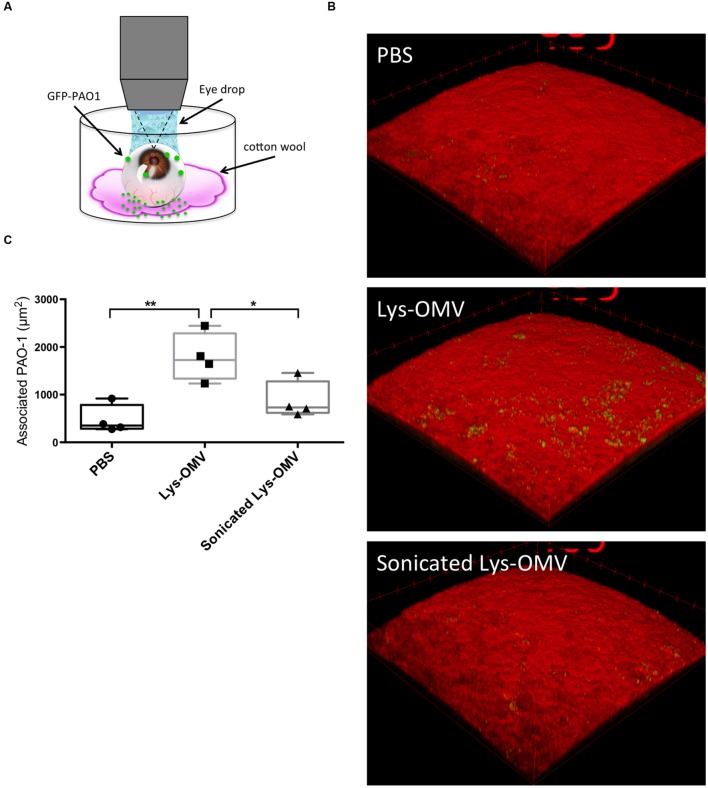
**(A)** Schematic representation of the experimental set up for imaging murine corneas *ex vivo*. **(B)** Confocal images of non-blotted murine corneas after 4 h *in vivo* pre-treatment with different OMV preparations or PBS control followed by 5 h *ex vivo* incubation of the enucleated eyes with ∼10^11^ CFU/ml *P. aeruginosa* PAO1-GFP. Representative 3D reconstructed images show greatly increased bacterial association with corneas treated with lysozyme-induced OMVs (second panel), which was significantly attenuated if the same OMVs were sonicated prior to pretreatment (third panel). **(C)** Quantification of *P. aeruginosa* associated with murine corneas under each condition. Data from four mice per group across four independent experiments are expressed as a median with upper and lower quartiles. ^∗^*P* < 0.03, ^∗∗^*P* < 0.004 (One-way ANOVA with Tukey’s multiple comparison test).

Given the cytotoxic impact of lysozyme-induced *P. aeruginosa* OMVs on mouse corneas, we explored their effect on *P. aeruginosa* interactions with the mouse corneal epithelium. One eye of mice was pretreated with one of the various OMV preparations, followed by incubation with ∼10^11^ CFU/ml *P. aeruginosa* PAO1-GFP for 5 h. Before OMVs were added, some corneas were blotted with tissue paper (Kimwipe^TM^). As described in our previous publication, this blotting process disrupts corneal epithelial barrier function against the small molecule fluorescein and increases susceptibility to *P. aeruginosa* adhesion (Tam et al., 2011). Eyeballs were imaged *ex vivo* using our published 3D quantitative confocal imaging methods (Tam et al., 2011), using an unpreserved eye drop between the objective and cornea to keep the surface moist (**Figure 8A**).

**FIGURE 9:**
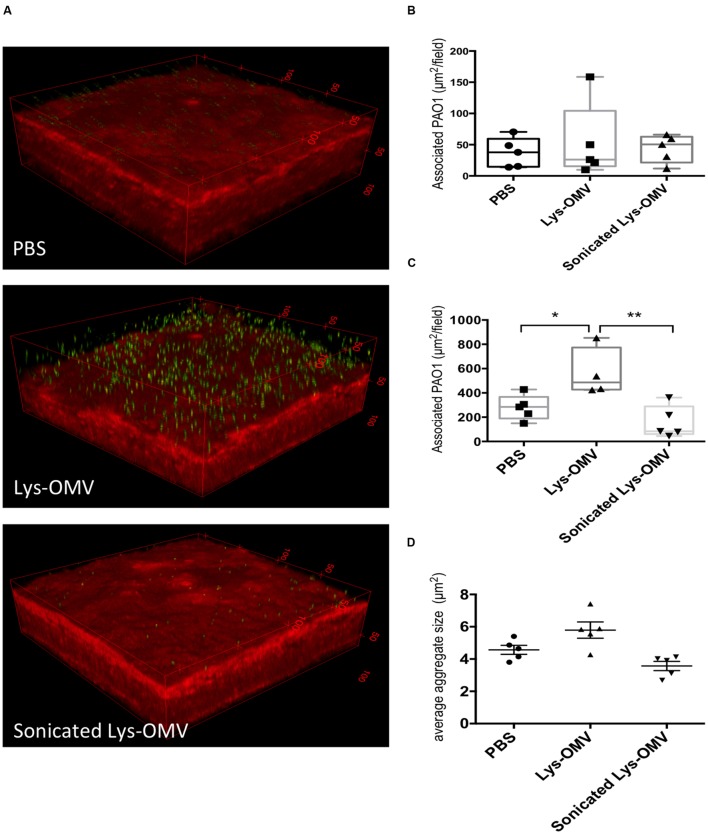
**(A)** Confocal images of murine corneas *ex vivo* after tissue-paper blotting and 1 h pre-treatment with different OMV preparations or PBS control followed by 5 h incubation with ∼10^11^ CFU/ml *P. aeruginosa* PAO1-GFP. Representative 3D reconstructed images show greatly increased bacterial association with blotted corneas also treated with lysozyme-induced OMVs (Lys-OMV, second panel), which did not occur if the same OMVs were sonicated prior to pretreatment (third panel). **(B,C)** Quantification of *P. aeruginosa* associated with healthy (non-blotted) murine corneas **(B)** or superficially injured (blotted) cornea **(C)** after 1 h *ex vivo* pre-incubation with PBS, Lysozyme-induced OMVs (Lys-OMV) and sonicated lysozyme-induced OMVs, followed by 5 h incubation with ∼10^11^ CFU/ml *P. aeruginosa* PAO1-GFP. **(D)** Average size (μm^2^) of *P. aeruginosa* aggregates in blotted eyes (measured with Image J) in the same experiment as **(C)**. Data shown are from 4 to 5 fields from each mouse cornea, and are expressed as a median with upper and lower quartiles for each condition. There was no significant difference between groups in non-blotted corneas, but association differences between groups were significant in blotted corneas **(C)** ^∗^*P* < 0.04, ^∗∗^*P* < 0.005, but not aggregate size **(D**; One-way ANOVA with Tukey’s multiple comparison test). In each instance, a representative experiment is shown of two experiments (non-blotted) and four experiments (blotted).

The results showed that even for previously healthy (non-blotted) corneas, there was a significant (∼4-fold) increase in the number of bacteria adhering to the corneal epithelium if the inoculum was added after corneal pre-treatment with intact lysozyme-induced OMVs for 4 h (**Figures 8B,C**). This increase was significant compared to both PBS and disrupted OMVs (*P* < 0.004 and *P* < 0.03, respectively). If corneas were superficially injured (blotted), only 1 h of OMV pretreatment was needed to promote a significant increase in bacterial adhesion (**Figures 9A,C**; Supplementary Figures S2A–C). However, 1 h OMV pretreatment of a healthy cornea (non-blotted) was insufficient to promote increased adhesion, even if the experiment was done *ex vivo* (**Figure 9B**). Thus, superficial injury rendered the corneas even more susceptible to OMV priming for *P. aeruginosa* adhesion.

For both blotted and non-blotted corneas, the adhesion promoting effect of OMVs required that OMVs were intact. Pre-exposure to sonicated OMVs produced effectively the same results as pre-exposure to control PBS (**Figures 8B,C** and **9A,C**; Supplementary Figures S2A–C) and uninduced OMVs (data not shown). While bacteria were bound to the epithelial surface in response to OMV priming, they did not appear to penetrate into deeper layers of the epithelium under any of the conditions examined.

Given the known contribution of OMVs to initiation of biofilms (Schooling and Beveridge, 2006; Schooling et al., 2009; Yonezawa et al., 2009) we also examined bacterial aggregates on the corneal epithelium (after 1 h OMV pre-treatment of blotted corneas). The average size and size distribution of *P. aeruginosa* aggregates on the murine corneal surface in each field was analyzed using Image J. No significant differences in *P. aeruginosa* aggregate size were observed when comparing OMV treatment with PBS controls (**Figure 9D**; Supplementary Figures S2D–F). Size distributions of *P. aeruginosa* aggregates in each field were also not significantly different among OMV treatment conditions (data not shown).

## Discussion

The results of this study showed that; (1) human tear fluid and purified lysozyme (but not corneal epithelial cell lysates, or lactoferrin) induce release of *P. aeruginosa*-derived OMVs that have a similar appearance and protein composition to each other, but not to biofilm harvested OMVs, (2) intact or disrupted OMVs are cytotoxic toward human corneal epithelial cells *in vitro* and to the mouse corneal epithelium *in vivo*, (3) intact but not sonicated OMVs increase *P. aeruginosa* adhesion to mouse corneas suggesting a mechanism that differs from their cytotoxic effects and that OMV fusion with the ocular surface is required, (4) superficial epithelial injury reduces the time required for OMVs to promote bacterial adhesion, and (5) OMV exposure increases expression of a myeloid cell marker at the corneal surface even in an otherwise healthy cornea *in vivo*. We also found that (6) *P. aeruginosa* laboratory and corneal isolates produce OMVs that vary in appearance and composition.

While OMV pre-exposure promoted adhesion, the adherent bacteria were not seen penetrating deeply through the epithelial multilayer, either *in vitro* or *in vivo*. This might be an artifact due of the relatively short timing or other conditions of these experiments. Alternatively, OMV exposure is not enough on its own to enable bacteria to penetrate. Another possibility is that defenses triggered by OMVs and/or the bacteria that adhere to the surface counter subsequent bacterial penetration in our assays. Candidate defenses could include antimicrobial peptide expression (Augustin et al., 2011), tightening of cell-to-cell junctions (Ulluwishewa et al., 2011; Yuki et al., 2011; Kuo et al., 2013), or NETs (Neutrophil Extracellular Traps) if they do occur. However, further studies are needed to support this hypothesis.

Outer membrane vesicles pretreatment reduced the number of bacteria in the apical compartment of the Transwell chambers in our *in vitro* experiments. Whatever the case, anything that promotes bacterial adherence to the epithelial surface could increase the probability that bacteria will eventually penetrate, particularly if other defenses are also compromised. In this regard, contact lens wear can inhibit innate defense responses of corneal epithelial cells, including antimicrobial peptide upregulation in response to *P. aeruginosa* (Maltseva et al., 2007).

Lysozyme-induced OMVs were cytotoxic to human corneal epithelial cells *in vitro* and mouse corneal epithelium *in vivo*. Since sonicated (disrupted) OMVs had the same toxicity as intact OMVs, the factor(s) involved may be carried externally on the OMV surface such as LPS as shown previously (Kadurugamuwa and Beveridge, 1995). While OMV cargo is mostly thought to be luminal, some factors have been associated with OMV surfaces, e.g., RNA and DNA (Dorward et al., 1989). Multiple virulence factors capable of cytotoxic effects are associated with *P. aeruginosa* OMVs including; protease (elastase), alkaline phosphatase, phospholipase C, and Cif (Kadurugamuwa and Beveridge, 1995; Bomberger et al., 2009), although these are delivered into the host cell cytosol requiring intact OMVs (Bomberger et al., 2009). In contrast to our data using human corneal epithelial cells, OMV cytotoxicity toward human airway epithelial cells required intact OMVs and more time (8 h, vs. 2 h in the present study; Bomberger et al., 2009). Interestingly, Cif was not required. Thus, the mechanisms of cytotoxicity observed for OMVs on corneal epithelial cells *in vitro* and *in vivo* may differ from those affecting airway cells *in vitro*. Differences in OMV effects may also reflect differences in other molecules commonly associated with *P. aeruginosa* OMVs, e.g., LPS, DNA (Kadurugamuwa and Beveridge, 1995) or PQS (Pseudomonas Quinolone Signal; Mashburn and Whiteley, 2005).

In addition to displaying more dead cells at their surface, previously healthy mouse corneas treated with OMVs *in vivo* for 4 h showed an increase in the total number of nuclei at their surface, and a greater number of cells that had polymorphic nuclei. This correlated with an increase in Ly6G/C positive cells at the corneal surface, strongly suggestive of myeloid cell recruitment after *in vivo* exposure to OMVs. Whether this happened directly in response to bacterial components within OMVs, or indirectly as result of cell injury, is to be determined. Also unclear is the significance of the result, considering that myeloid cells play desirable roles in bacterial clearance and wound healing (Hanlon et al., 2014; Shan et al., 2014), but can also contribute to inflammation and tissue disruption. Curiously, many of the Ly6G/C positive cells were similar in size and shape to corneal epithelial cells (**Figure 7A**), another finding that will require further investigation.

Contrasting with the cytotoxic effects of OMVs to the corneal epithelium, the capacity of OMVs to prime corneal epithelium for bacterial adhesion required that OMVs were intact. That finding suggests more than one mechanism of action for OMVs at the ocular surface, and that OMV delivery of their intravesicular cargo onto or into host epithelial cells is required for promoting adhesion.

Mechanisms by which OMVs might enhance susceptibility to adhesion could include cytosolic delivery of Cif (Bomberger et al., 2009) which promotes CFTR degradation in lysosomes (Bomberger et al., 2011). Loss of CFTR decreases *P. aeruginosa* internalization by airway and corneal epithelial cells (Pier et al., 1996; Zaidi et al., 1996) which could cause increased levels of surface-associated bacteria. More directly, OMV delivery of proteases and/or phospholipase C could further damage murine corneal epithelial surfaces to create more sites for bacterial binding. OMV-mediated increases in *P. aeruginosa* association with the cornea could involve facilitation of bacterial aggregation and biofilm formation as shown for *P. putida* (Baumgarten et al., 2012), although our data did not show any differences in the size or shape of aggregates adhering to the cornea after OMV exposure.

Why the superficially injured cornea was primed more quickly for bacterial adhesion is not clear. Possibilities include that deeper layer cells exposed by blotting were more susceptible, that blotting removed extracellular factors protective against the effects of OMVs (e.g., mucins), or that the mechanism differs entirely for healthy and superficially injured corneas. For example, recruitment of Ly6G/C positive cells seen at the 4 h time point when previously healthy corneas became susceptible to adhesion might contribute. Indeed, the diffuse NucGreen staining (not confined to the nucleus) upon bacterial challenge is suggestive of release of chromosomal DNA from dead or dying cells (Supplementary Figure S1). This phenomenon could indicate potential generation of NETs with longer OMV exposure (Brinkmann et al., 2004; Shan et al., 2014).

Lysozyme-induced OMVs showed the same banding pattern as tear-induced OMVs in SDS-PAGE, and were similar in appearance by TEM. While this suggests that it is the lysozyme in tear fluid that triggers OMVs, we cannot rule out the possibility that other tear components participate. Indeed, tear fluid contains multiple other factors that could be involved, such as cationic antimicrobial peptides (e.g., α-defensins and bacterial permeability increasing protein; McDermott et al., 2003; McDermott, 2009), neutrophils and their products (Sakata et al., 1997; Zhou et al., 2004; Peuravuori et al., 2006), secretory IgA and SP-D (Masinick et al., 1997; Ni et al., 2005).

While more research will be needed to determine the mechanism(s) of OMV induction from *P. aeruginosa* by tear fluid and lysozyme, physical mechanisms of membrane disruption as shown for other OMV inducers are likely to be involved (Schertzer and Whiteley, 2013). Lysozyme is cationic and disrupts cell wall peptidoglycan via its muraminidase activity, but also disrupts outer and cytoplasmic membranes of Gram-negative bacteria (Pellegrini et al., 1992; Derde et al., 2015). Of possible relevance, the triggering of *P. aeruginosa* OMVs by gentamicin was shown to involve its cationic properties via outer membrane disruption (Kadurugamuwa and Beveridge, 1997). While the mechanism by which lysozyme generates OMVs might be similar, lactoferrin is another cationic agent with bacteriostatic activity against *P. aeruginosa* and it did not induce OMV release in our hands.

Extensive differences in OMV composition were suggested by SDS-PAGE variations among TSA-grown OMVs released by different *P. aeruginosa* strains, and between tear or lysozyme-induced OMVs versus TSA-grown OMVs for strain PAO1. Growth-related OMV differences could potentially contribute toward variability between different *P. aeruginosa* isolates when interacting with the host. For example, under different experimental conditions to the present study, cytotoxic, but not invasive, isolates were found to use OMVs to escape neutrophil extracellular traps (Shan et al., 2014), yet in the present study, cytotoxic and invasive isolates could not be distinguished by TSA-grown OMV patterns. Tear or lysozyme-induced OMVs also appeared more complex than TSA-grown OMVs for strain PAO1. Further studies, including proteomic and lipidomic analysis, will be required to delineate differences in OMVs between stress-induced (tear fluid/lysozyme) and TSA-grown OMVs in *P. aeruginosa*, and relate them to functional differences in their interaction with epithelial cells.

Taken together, these data may contribute another piece of the puzzle as to how contact lens wear predisposes to corneal pathology. Normally, bacteria inoculated into the eye are quickly cleared, even if introduced in large numbers. However, *P. aeruginosa* is able to form biofilms on the posterior surfaces of contact lenses during wear, at least in rats. On the back of a worn lens, they would be exposed to sequestered tear fluid and tear-derived factors that adsorb onto the lens [including lysozyme (Mannucci et al., 1985; Hall et al., 2014)] for extended periods of time – potentially long enough to trigger OMV production. OMVs could also be produced during disinfection in lens storage cases, and transferred into the eye during lens insertion. Due to the lack of tear exchange behind a soft contact lens, OMVs trapped in that location could disrupt corneal epithelial barrier function against bacteria. The latter would be in addition to whatever compromise results directly from lens wear on a cornea, and any live bacteria present. Since the data also suggest that *P. aeruginosa* OMVs can trigger Ly6G/C expression on the surface of the cornea – suggesting inflammatory cells are present – the results of this study could also relate to development of “sterile” inflammatory responses during contact lens wear. At present, the above scenario represents a theoretical model for OMV involvement in the pathogenesis of contact lens-related *P. aeruginosa* keratitis. However, if substantiated by data from future studies, new lens care solutions or contact lenses could be developed that minimize OMV induction by *P. aeruginosa*, and other Gram-negative bacteria, and protect the corneal epithelium from OMV mediated compromise.

## Author Contributions

MM, DE, MG, JK, and SF designed the experiments. MM performed the experiments. MM, DE, MG, JK, and SF analyzed the experiments. MM, DE, and SF wrote the manuscript.

## Conflict of Interest Statement

The authors declare that the research was conducted in the absence of any commercial or financial relationships that could be construed as a potential conflict of interest. Dr. Fleiszig was a paid consultant with Allergan Inc. when this work was performed. That activity was unrelated to the work presented in the manuscript. Dr. Gabriel and Dr. Kadurugamuwa are paid employees of Alcon Research, Ltd.
