# Supplementary material for: Pseudomonas aeruginosa Outer Membrane Vesicles Triggered by Human Mucosal Fluid and Lysozyme Can Prime Host Tissue Surfaces for Bacterial Adhesion
Source: Front Microbiol. 2016 Jun 3;7:871. doi: 10.3389/fmicb.2016.00871 (PMC4891360; doi:10.3389/fmicb.2016.00871)
Supplement: Supplementary file 1 [file Data_Sheet_1.DOCX]

Supplementary Material

*Pseudomonas aeruginosa* Outer Membrane Vesicles Triggered by Human Mucosal Fluid and Lysozyme Can Prime Host Tissue Surfaces for Bacterial Adhesion

Matteo M. E. Metruccio, David J. Evans, Manal M. Gabriel, Jagath L. Kadurugamuwa, Suzanne M. J. Fleiszig*

*** Correspondence:** Suzanne M. J. Fleiszig: fleiszig@berkeley.edu

## Supplementary Figures

**Supplemental Figure 1.** Confocal images of non-blotted murine corneas after 4 h *in vivo* pre-treatment with lysozyme-induced OMV followed by 5 h *ex vivo* incubation of the enucleated eyes with ~10^11^ CFU/mL *P. aeruginosa* PAO1-dTomato. Whole eyeballs were then incubated with NucBlue and NucGreen for 30 min prior to imaging. Maximum intensity projection of blue channel (all nuclei), green channel (dead cells), red channel (PAO1-dTomato) and merge is shown. White arrows show regions in which the green dye appear diffuse, suggesting free DNA released from dead cells, with associated bacteria.

**Supplemental Figure 2.** Quantification of *P. aeruginosa* associated with superficially-injured (blotted) corneas (A, B and C) and average size of bacterial aggregates (D, E, and F) after 1 h ex vivo pre-incubation with PBS, lysozyme-induced OMVs (Lys-OMV) and sonicated lysozyme-induced OMVs, followed by 5 h incubation with ~10^11^ CFU/mL *P. aeruginosa* PAO1-GFP for 3 independent experiments (A and D, B and E, C and F). Data shown are from 4 to 5 fields from each mouse cornea, and are expressed as a median with upper and lower quartiles for each condition. Pre-incubation with Lysozyme-induced OMVs resulted in significantly more bacteria associated with murine corneas in all replicates * P < 0.05, ** P < 0.01, **** P < 0.0001 (One-way ANOVA with Tukey's multiple comparison test). Aggregate sizes were not significantly different between groups.
