# Supplementary figures and images for: Pseudomonas aeruginosa Outer Membrane Vesicles Triggered by Human Mucosal Fluid and Lysozyme Can Prime Host Tissue Surfaces for Bacterial Adhesion
Source: Front Microbiol. 2016 Jun 3;7:871. doi: 10.3389/fmicb.2016.00871 (PMC4891360; doi:10.3389/fmicb.2016.00871)

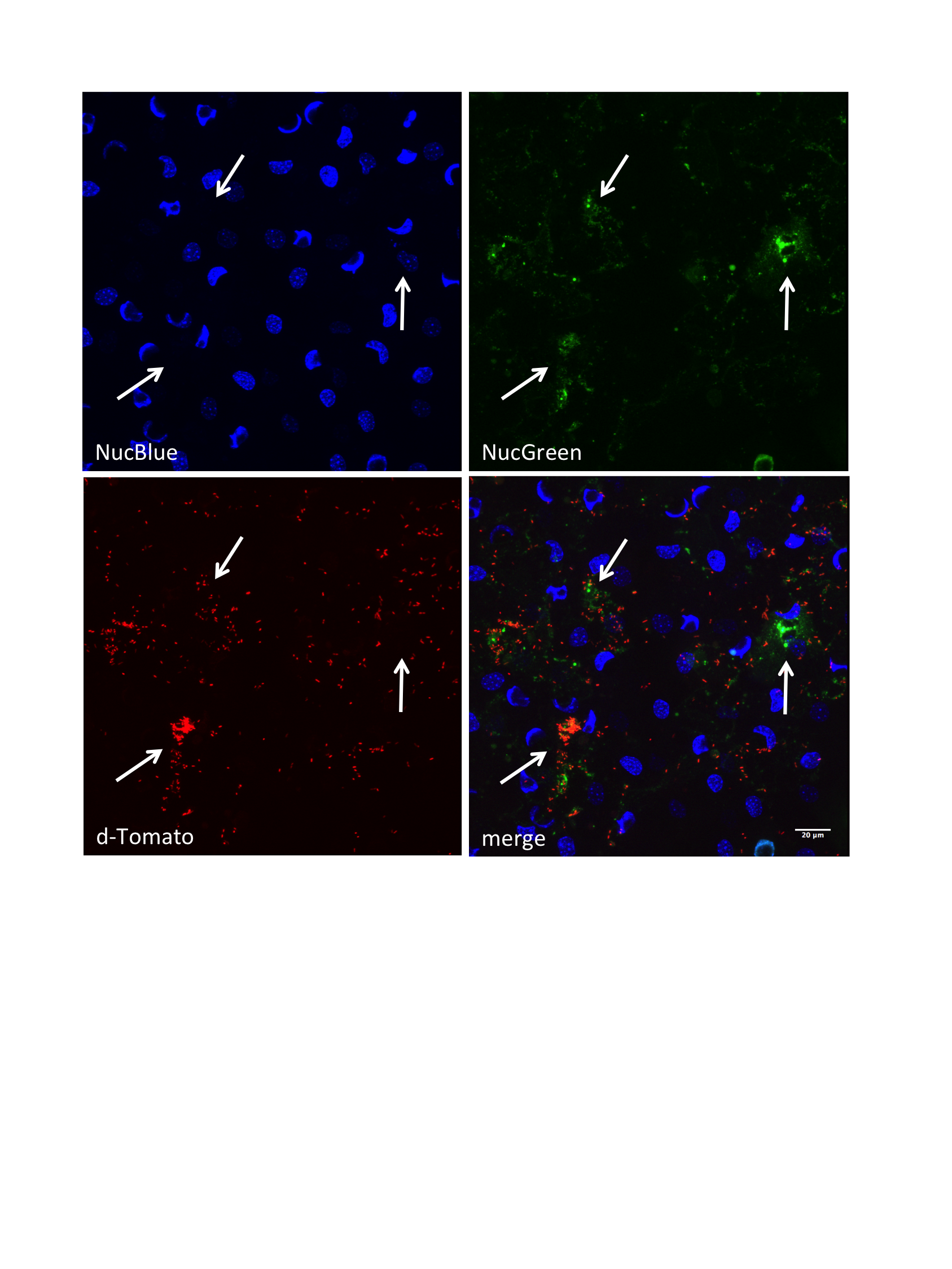

Supplement: Supplementary file 2 [file Image_1.TIF]

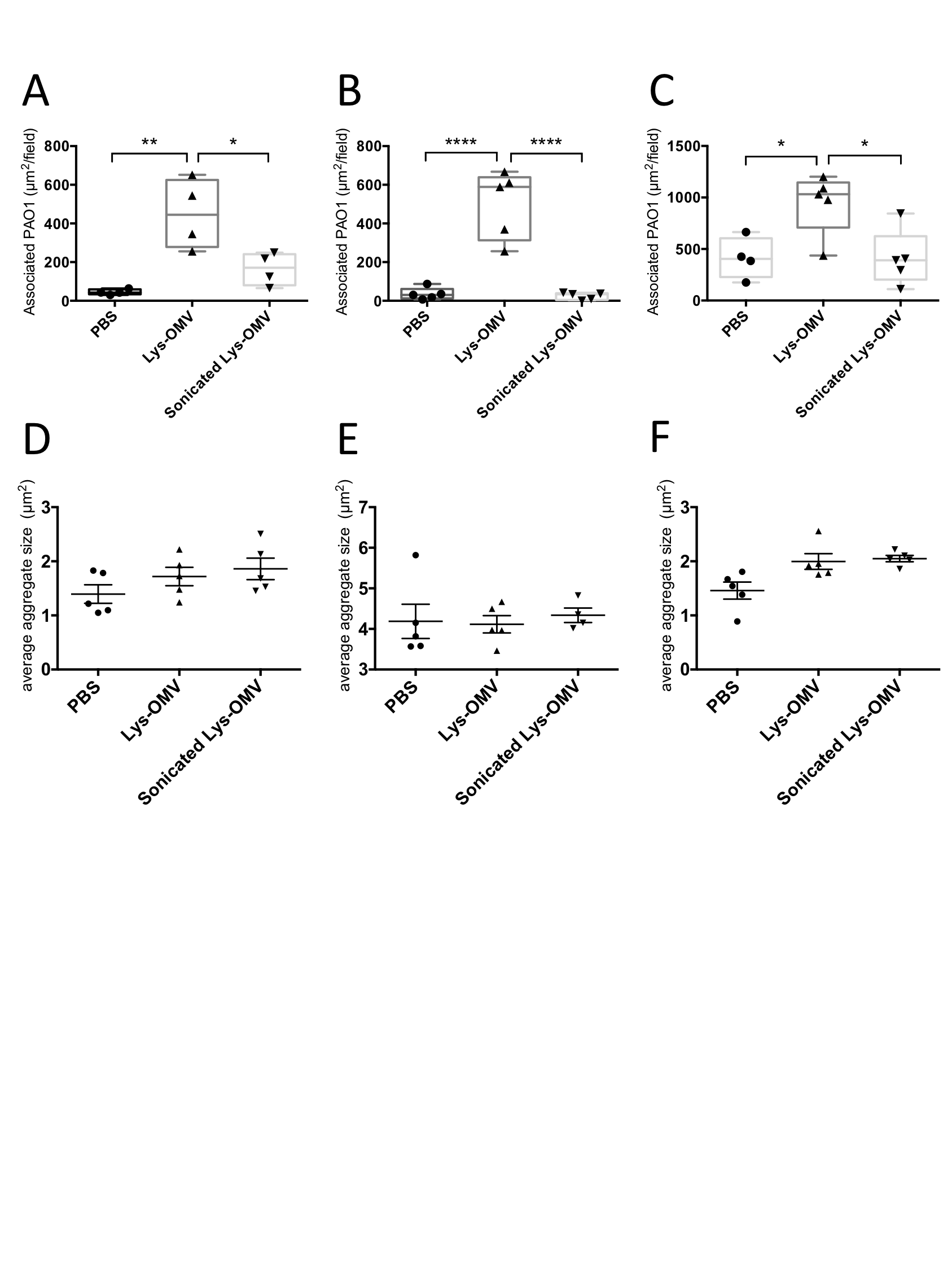

Supplement: Supplementary file 3 [file Image_2.TIF]
